# Effect of Maternal Obesity on Fetal Growth and Expression of Placental Fatty Acid Transporters

**DOI:** 10.4274/jcrpe.4510

**Published:** 2017-12-15

**Authors:** Kui Ye, Li Li, Dan Zhang, Yi Li, Hai-Qing Wang, Han-Lin Lai, Chuan-Lai Hu

**Affiliations:** 1 Anhui Medical University School of Public Health, Department of Nutrition and Food Hygiene, Anhui, China; 2 Lujiang Center for Disease Control and Prevention, Department of Public Health, Anhui, China; 3 Anhui Provincial Hospital, Clinic of Clinical Nutrition, Anhui, China

**Keywords:** Obesity, placenta, nutrient transport, high-fat diet, intrauterine growth

## Abstract

**Objective:**

To explore the effects of maternal high-fat (HF) diet-induced obesity on fetal growth and the expression of placental nutrient transporters.

**Methods:**

Maternal obesity was established in rats by 8 weeks of pre-pregnancy fed HF diet, while rats in the control group were fed normal (CON) diet. Diet-induced obesity (DIO) rats and diet-induced obesity-resistant (DIR) rats were selected according to body weight gain over this period. After copulation, the CON rats were divided into two groups: switched to HF diet (CON-HF group) or maintained on the CON diet (CON-CON group). The DIO rats and DIR rats were maintained on the HF diet throughout pregnancy. Pregnant rats were euthanized at day 21 gestation, fetal and placental weights were recorded, and placental tissue was collected. Reverse transcription-polymerase chain reaction was used to determine mRNA expression of placental nutrient transporters. Protein expression was determined by Western blot.

**Results:**

Average fetal weight of DIO dams was reduced by 6.9%, and the placentas of CON-HF and DIO dams were significantly heavier than the placentas of CON-CON and DIR dams at day 21 of gestation (p<0.05). The fetal/placental weight ratio of DIO dams was significantly reduced compared with the fetal/placental weight ratio of CON-CON dams (p<0.05). The mRNA expression of GLUT-1 and SNAT-2 were not significantly different between groups. The mRNA and protein expression levels of CD36, FATP-1, and FATP-4 in DIO dams were decreased significantly (p<0.05).

**Conclusion:**

Maternal obesity induced by a HF diet led to intrauterine growth retardation and down-regulated the expression of placental fatty acid transporters.

**What is already known on this topic?**

Previous studies have investigated the association between maternal obesity and placental nutrient transporters, but results have been inconsistent.

**What this study adds?**

One of the most fundamental questions arising from the study is how diet-induced obesity and diet-induced obesity-resistant dams and their offspring differ in terms of their respective metabolic response to a high-fat diet.

## INTRODUCTION

Maternal obesity may lead to serious health complications for mother and fetus. It increases the incidence of maternal pregnancy complications such as gestational diabetes and preeclampsia (1,2,3,4). In addition to maternal health complications, there is also a considerable increase in the risk of fetal complications. These include stillbirth (3,5,6), spontaneous abortions (5) and fetal asphyxia (7), as well as increased risk of delivery of small for gestational age or large for gestational age babies (8,9,10). In the long-term, the offspring of obese mothers have a higher risk of obesity in adult life, and this is likely due to the combined effects of genetics and environment. Given these factors, understanding how maternal obesity might have an impact on offspring health is of major public health importance.

Pregnancy is a critical period of physiological change for both the mother and the fetus. The placenta, the interface between the maternal and fetal blood circulations (11), is responsible for the maternal-to-fetal transfer of nutrients which are essential for fetal growth and development. Fetal growth is directly related to maternal nutrient availability and the placental ability to transport these nutrients from maternal circulation to the fetus. Glucose, amino acids, and fatty acids are essential macronutrients for adequate fetal growth. All of these traverse the placental syncytiotrophoblasts (SCTB) mediated by specific transporters. Placental glucose transport occurs by facilitated diffusion along a concentration gradient through members of the glucose transporter (GLUT) family (12,13). Amino acid transport across the human placenta is a complex process because more than 20 different amino acid transporters with overlapping specificities are expressed in the SCTB. For example, system A is a sodium-dependent accumulative transport system which mediates the uptake of neutral amino acids (both essential and nonessential) with short and unbranched side chains, mostly L-alanine, glycine, L-serine, L-methionine, and L-glutamine (14,15). In recent years, a gene family of sodium-coupled neutral amino acid transporters [(SNAT); (SCL38 gene family)] coding for proteins that possess the classically described system A transport activities (in terms of their functional properties and patterns of regulation) has been cloned (16). SNAT-2 is widely expressed in rat tissues, and its mRNA concentration increases in cultured cells during adaptive regulation or with the addition of cAMP (17). The proteins associated with fatty acid transport include fatty acid transport proteins (FATP), fatty acid translocase (FAT/CD36), plasma membrane fatty acid binding protein (FABPpm), and other FABP (18,19). FATPs, six members (FATPs1-6), are integral membrane proteins that are of importance for the uptake of long-chain fatty acids (18). Five members (FATPs1-4, and 6) have been identified in placental trophoblasts (18). FATP-1 and FATP-4 have been frequently studied in placental tissue as their expression correlates with docosahexaenoic acid levels in maternal plasma, cord blood, and placental phospholipids, suggesting an important role in the transfer of long-chain polyunsaturated fatty acids (20). Of note, previous studies have investigated the association between maternal obesity and placental nutrient transporters, but results have been inconsistent. Reynolds et al (21) have reported that maternal high-fat (HF) consumption induces sex-specific nutrient transport in the rat placenta (21). In this study, maternal HF consumption was found to be associated with increased placental CD36 mRNA expression only in female placentas, and placental GLUT1, GLUT4, and SNAT2 mRNA expression significantly increased only in HF male placentas. This sex difference may be driven by sexually dimorphic placental alterations which occur as a result of maternal gestational diets. Farley et al (22) reported that maternal obesity was accompanied by decreased placental SNAT activity although the offspring grew normally. In a Canadian study in humans, it was found that maternal obesity was associated with increased placental CD36 mRNA and protein expression and decreased FATP-4 mRNA and protein and FABP3 protein expression (23). However, Zhu et al (24), working with sheep, showed that maternal obesity enhanced the mRNA expression and protein content of FATPs in the placenta. These complex and conflicting results raise significant doubt as to whether placental nutrient transport is associated with maternal obesity. Therefore, in this study, we developed a model of maternal obesity in which rats are fed a HF diet and used this model to determine how maternal obesity influenced fetal growth, placental weight, as well as placental gene and protein expression of nutrient transporters.

## METHODS

All animal procedures were approved by the Animal Ethics Committee of Anhui Medical University (approval number: 20131188). Female Sprague-Dawley rats, 6 weeks old, were obtained from the Experimental Animal Center of Anhui Medical University. The rats were maintained in controlled temperature (23-25 °C), light (12-hr light - dark cycle), and humidity (55±5%) conditions with access to food and water ad libitum. Subsequent to a seven day adaptation period, animals were given ad libitum access to either a control (CON; n=40) diet of standard rodent chow (3.435 kcal/g; 12% energy as fat, Jiangsu Xie Tong Biological Engineering Co., Ltd.) or a HF; (n=53) diet which contained 70% normal chow, 10% lard, 1% cholesterol, 3% casein, 10% egg yolk powder, and 6% whole-milk powder (4.487 kcal/g; 45% energy as fat, Jiangsu Xie Tong Biological Engineering Co., Ltd.). Body weight of each dam was monitored weekly. After 7 weeks of feeding, rats fed the HF diet exhibited varying somatic weight change in response to the diet. According to the approach undertaken in previous studies (25,26), 18 rats with the highest body weight gain were designated as diet-induced obesity (DIO) rats, while 18 rats with lowest body weight gain were designated as diet-induced obesity-resistant (DIR) rats. During the 8th week, the food intakes of the CON, DIO, and DIR rats were measured. At the end of the 8th week, the CON, DIO, and DIR rats were mated with age-matched Sprague-Dawley male rats fed the CON diet. Copulation was confirmed by the presence of sperm in a vaginal flush; the day of copulation was designated gestational day (GD) 0. After copulation, the CON rats were divided into two group: half were switched to the HF diet (CON-HF group, n=10), while half were maintained on the CON diet (CON-CON group, n=10). The DIO (n=10) rats and DIR (n=10) rats were maintained on the HF diet throughout pregnancy. Food intakes during pregnancy were measured. Body weights were monitored weekly. At the end of GD21, all rats were fasted overnight prior to being euthanized with chloral hydrate which was also used in previous studies (27,28,29). Blood samples of rats were obtained from the common abdominal aorta for measurements of insulin levels. Fetuses in each litter were counted and weighed, and then crown-rump length was measured. Fetal blood samples were also collected for measurements of insulin level. Maternal and fetal blood glucose levels were measured immediately using an Accu-Chek blood glucose monitor (Accu-Chek; Roche Diagnostics, Mannheim, Germany) (30,31). Serum insulin level was determined by radioimmunoassay (Beijing North Biotechnology Research Institute, Beijing, China). Placental weights were recorded, and placentas were snap frozen in liquid nitrogen and stored at -80 °C until further analysis.

### Isolation of Total RNA and Real-time Polymerase Chain Reaction

Total RNA was extracted from placental tissues, and real-time polymerase chain reaction (rt-PCR) performed as previously described (32). Total RNA was extracted using TRI reagent (Molecular Research Center). Ribonuclease-free deoxyribonuclease-treated total RNA (1.0 μg) was reverse-transcribed with Aves myeloblast leukemia virus reverse transcriptase (Promega). RT-PCR was performed with a LightCycler 480 SYBR Green I kit (Roche Diagnostics GmbH) using gene-specific primers as listed in Table 1. Specific primers were synthesized by Shanghai Sangon Biological Engineering Technology (Shanghai, China). The amplification reactions were carried out on a LightCycler 480 Instrument (Roche Diagnostics GmbH) with an initial hold step (95 °C for 5 minutes) and 50 cycles of a three-step PCR (95 °C for 15 seconds, 60 °C for 15 seconds, 72 °C for 30 seconds). The comparative cycle threshold method was used to determine the amount of target, normalized to an endogenous reference (GAPDH) and relative to a calibrator using the LightCycler 480 software (version 1.5.0; Roche).

### Western Blot

Total lysate from rat placentas was prepared by homogenizing 50 mg placenta tissue in 300 μL lysis buffer (50 mM Tris-HCl, pH 7.4, 1 mM EDTA, 150 mM NaCl, 0.1% sodium dodecyl sulfate, 1% Triton X-100, 1% sodium deoxycholate, 1 mM phenylmethylsulfonyl fluoride) supplemented with a cocktail of protease inhibitors (Roche). Protein concentrations were determined using bicinchoninic acid (BCA) protein assay reagents (Pierce, Rockford, IL) according to manufacturer’s instructions. The levels of CD36, FATP1, and FATP4 in placental tissue were quantified using Western blot, as previously described (32). Briefly, an amount of protein (40 ~ 80 μg) was separated electrophoretically by SDS-PAGE and transferred to a polyvinylidene fluoride membrane. Membranes were blocked in 5% non-fat milk in tris buffered saline (TBST) (137 mM NaCl, 2.7 mM KCl, 25 mM Tris-Cl, pH 8.0) supplemented with 0.1% Tween-20 overnight at 4 °C. The membranes were incubated for 2 hours with the following antibodies: CD36 (1:500; Abcam Inc, Cambridge, MA), FATP1 (1:500; Abcam Inc, Cambridge, MA), and FATP4 (1:1000; Abcam Inc, Cambridge, MA). β-actin (1:2000; Santa Cruz Biotechnologies, CA, USA) was used as a loading control. After being washed in TBST containing 0.05% Tween-20 four times for 10 min each, the membranes were incubated with secondary antibody (goat anti-rabbit IgG or goat anti-mouse IgG; both from Santa Cruz Biotechnologies, CA, USA) for 2 hours. The membranes were then washed for four times in TBST containing 0.05% Tween-20 for 10 min each. Finally, enhanced chemiluminescence solution (ECL kit; Pierce Biotechnology, USA) was added, and Fine-do X6 visualizer was used for the photographing (Tanon, Shanghai, China).

### Statistical Analysis

All statistical analysis was performed using the SPSS 13.0 software (SPSS Inc, Chicago, IL). For animal experiments, the litter was considered the unit for statistical analysis among different groups. For fetal weight and crown-rump length, the means were calculated per litter. All values are expressed as the mean ± standard deviation (SD). Comparisons between CON and HF groups were compared using two independent sample t-test. One-way ANOVA was used to determine differences among three or more groups and further comparison between two groups was assessed with least significant difference (LSD) post hoc test. A p-value <0.05 was considered statistically significant.

## RESULTS

### Effect of HF Diet on Body Weight, Food Intake, Energy Intake, Fasted Blood Glucose, and Serum Insulin of Dams

**Figure 1:**
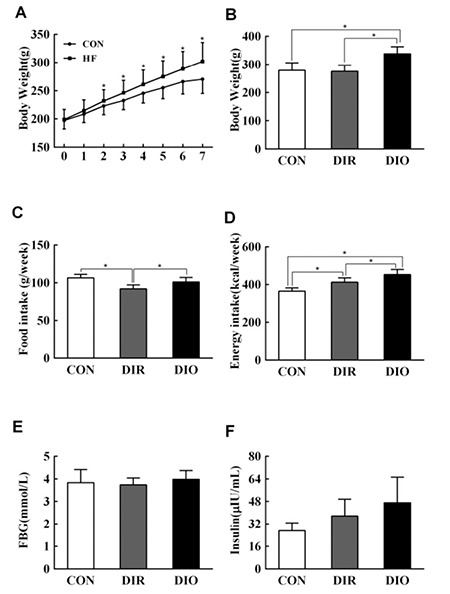
Body weight, food intake, energy intake, fasted blood glucose, and serum insulin analysis in rats. A) body weight in rats that were fed either a control or high-fat diet, B) body weight at 8 weeks, C) food intake during the 8th week, D) energy intake during the 8th week, E) fasting blood glucose at 8 weeks, F) serum insulin after 8 weeks feeding Data are mean + standard deviation *Indicates p<0.05 FBG: fasting blood glucose, DIR: diet-induced obesity-resistant, DIO: diet-induced obesity, HF: high-fat.

After two weeks, dams fed HF diet were significantly heavier than CON dams (t=2.254, p=0.029, Figure 1A). After 7 weeks of feeding, there was a significant difference in body weight among CON rats, DIR rats, and DIO rats (F=39.864, p<0.001, Figure 1B); the weight of DIO rats was significantly higher than that of CON rats (21.2%, p<0.05). There was a significant difference in food intake and energy intake among the three groups (F=7.477, p=0.002, Figure 1C; F=28.412, p<0.001, Figure 1D). However, there was no difference in weight between CON and DIR rats at this time. There was no significant difference in fasted blood glucose or serum insulin among the three groups (Figure 1E and 1F).

### Weight Gain, Food Intake, and Energy Intake During Pregnancy, Fasted Blood Glucose and Serum Insulin of Dams at GD21

**Figure 2:**
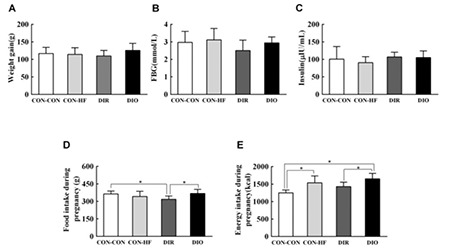
Weight gain, food intake, energy intake during pregnancy, and fasting blood glucose and serum insulin at gestational day 21. A) weight gain, B) fasting blood glucose, C) serum insulin, D) food intake, E) energy intake during pregnancy (n=10 per group) Data are mean ± standard deviation *Indicates p<0.05 FBG: fasting blood glucose, DIR: diet-induced obesity-resistant, DIO: diet-induced obesity, HF: high-fat.

There was no significant difference in weight gain during pregnancy among the four groups (CON-CON: 116.78±17.58 g, CON-HF: 114.21±18.93 g, DIR: 109.77±15.93 g, DIO: 125.69±20.24 g; F=1.353, p=0.273; Figure 2A). However, fasting blood glucose and serum insulin levels at GD21 were not significantly different (Figure 2B, 2C). There were significant differences in food intake and energy intake during pregnancy among the four groups (F=3.547, p=0.028, Figure 2D; F=9.848, p<0.001, Figure 2E). The food intake of DIR rats was less than that of CON-CON rats and DIO rats during pregnancy (Figure 2D). The energy intake during pregnancy was more in DIO rats and CON-HF rats than in CON-CON rats and DIR rats (Figure 2E).

### Effect of Maternal Obesity on Fetal Growth, Placental Weight, and Fetal to Placental Weight Ratio

**Figure 3:**
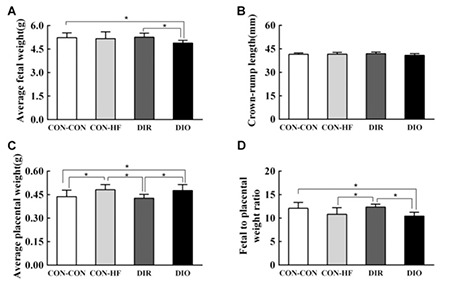
Effect of maternal obesity on fetal growth, placental weight, and fetal to placental weight ratio. A) average fetal weight, B) crown-rump length, C) placental weight, D) fetal to placental weight ratio. n=10 per group Data are mean ± standard deviation *Indicates p<0.05 DIR: diet-induced obesity-resistant, DIO: diet-induced obesity, HF: high-fat.

Average fetal weight of DIO dams was reduced by 6.9% (DIO: 4.88±0.17 g, CON-CON: 5.23±0.31 g, p<0.05; Figure 3A), while the average fetal weight of DIR dams and CON-HF dams was similar to those of CON-CON dams. Mean fetal weight was significantly lower in DIO offspring compared with DIR offspring. However, there was no significant difference in offspring crown-rump length between the four groups (Figure 3B). At GD21, the placentas of CON-HF and DIO dams were heavier than those of CON-CON and DIR dams (Figure 3C). Figure 3D shows that the fetal to placental weight ratio of DIO dams was significantly lower compared with the fetal to placental weight ratio of CON-CON and DIR dams.

### Effect of Maternal Obesity on Placental mRNA and Protein Expression of Nutrient Transporter

**Figure 4:**
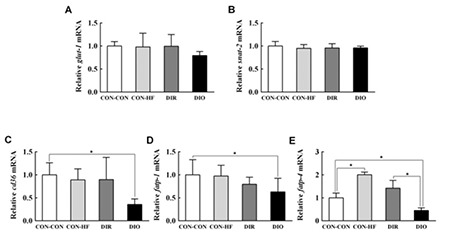
Effect of maternal obesity on placental mRNA expression of nutrient transporters. A) GLUT-1, B) SNAT-2, C) CD36, D) FATP-1, E) FATP-4, n=6 per group. Data are mean ± standard deviation *Indicates p<0.05. DIR: diet-induced obesity-resistant, DIO: diet-induced obesity, HF: high-fat

**Figure 5:**
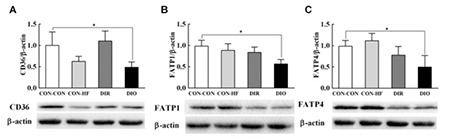
Effect of maternal obesity on placental fatty acid transporters protein expression. A) CD36, B) FATP-1, C) FATP-4. n=6 per group Data are mean + standard deviation *Indicates p<0.05 DIR: diet-induced obesity-resistant, DIO: diet-induced obesity, HF: high-fat

The mRNA expression of GLUT-1 and SNAT-2 was not significantly different between the groups (Figure 4A and 4B). The mRNA expression of placental fatty acid transporters CD36, FATP-1, and FATP-4 in DIO dams was decreased by 35.7%, 62.9%, and 45.0%, respectively (Figure 4C, 4D, 4E). FATP-4 mRNA expression in DIO dams was significantly reduced compared with that in DIR dams. The mRNA expression of CD36, FATP-1, and FATP-4 in the CON-HF group was not significantly reduced compared with that in CON-CON group. As shown in Figure 5, placental fatty acid transporter protein expression in DIO dams was significantly decreased compared with that in CON-CON dams. Protein expression of CD36, FATP-1, and FATP-4 in DIO dams was decreased by 48.7%, 57.2%, and 50.4%, respectively (Figure 5A, 5B, 5C). Protein expression of CD36, FATP-1, and FATP-4 was not significantly reduced in CON-HF group compared with the CON-CON dams.

## DISCUSSION

We report the effect of maternal HF, DIO on fetal growth and placental nutrient transport. We found correlation between maternal HF, DIO and reduced fetal growth. However, in animals resistant to induced obesity, no reduction in fetal weight was observed. Meanwhile, maternal HF, DIO down-regulated the expression of placental fatty acid transporters (CD36, FATP-1 and FATP-4).

Obesity results from complex interactions of environmental and genetic components which facilitate the development of an obese phenotype (33). Diet is the most important environmental factor leading to obesity, and models of HF DIO are commonly used in studies. After feeding with a high fat diet, rats exhibited different phenotypes in response to the diet. Therefore, we selected the DIO and DIR rats according to weight gain after 7 weeks of HF feeding. We found that food intake and energy intake of DIR rats were lower than those of DIO rats, consistent with previous reports (34). Liu et al (35) reported that DIR rat has the ability to sense and respond to energy imbalance accurately, while the ability in DIO is blunted. Despite having the same feeding conditions as the DIO group, DIR rats are sensitive to the energy balance system and can adjust their energy expenditure to maintain a normal weight depending on the level of energy intake.

Previous studies have investigated the association between maternal obesity and fetal growth, but results have been contradictory. Increased risks for both fetal macrosomia and intrauterine growth restriction (IUGR) have been reported (8,9,10,36,37). In the current study, fetal weight of DIO dams was significantly reduced, and a clear association between maternal obesity and an increased the risk for IUGR were shown. Furthermore, DIO dams had normal blood glucose and serum insulin levels in our study, suggesting the absence of gestational diabetes mellitus despite the obese phenotype. In previous studies, obese mothers often developed abnormal glucose homeostasis (10,38). Thus, reports show inconsistencies which may be due to study design (contents of HF diet, time of HF diet feeding), degree of obesity in subjects, or species difference. Our results also demonstrate that fetal growth is different in DIO and DIR dams. Despite having the same feeding conditions as the DIO group, the offspring of DIR rats seem to be healthier compared with DIO offspring, consistent with previous reports (34). In a future study, the focus will be on the issue of how the DIR rats are protected from the deleterious effects of a HF diet.

The fetal to placental weight ratio has been considered to be a marker of placental nutrient transporter efficiency (39,40). A lower fetal to placental weight ratio may indicate below average placental nutrient transport efficiency. In our study, fetal to placental weight ratio was lower in DIO dams indicating that placental nutrient transport efficiency in DIO dams was decreased.

Fetal growth is mainly dependent on fetal nutrient availability, which is determined by the capacity of the placenta to transport nutrients. The transport of placental fatty acids is critical for fetal growth, particularly in late gestation (41). A previous study found that maternal obesity was associated with decreased FATP-4 mRNA and protein expression, whereas CD36 expression was increased (23). We found that the fatty acid transporters (CD36, FATP-1, and FATP-4) mRNA and protein expression were down-regulated in the DIO placenta. Thus, maternal obesity was associated with decreased placental fatty acid transporter mRNA and protein expression. It has been reported that several placental transport functions are altered in pregnancies complicated by IUGR (42). Placental fatty acid transporters may have an important role to play in the process of IUGR induced by maternal obesity. Our results are inconsistent with previously reported results (21,22,23,24), which may be due to species difference (rat, sheep, human, etc.), degree of obesity in subjects, study design, or gestational age studied (mid-gestation, late pregnancy, delivery, etc.).

### Study Limitations

There are several limitations of the current study. Due to the nature of our obesity model, we are limited by the number of rats in our study, which left us underpowered to thoroughly assess differences between male and female fetuses. Thus, though we did not detect any trends, we cannot conclusively rule out the effect of fetal sex on these relationships. In addition, some metabolic factors (i.e. leptin, inflammation, lipids and fatty acids levels) in maternal circulation were not measured in current study. We are currently unable to ascertain how maternal obesity affected placental fatty acid transport. Further studies will be designed to explore the mechanism.

## CONCLUSION

In summary, the current study indicates that maternal HF, DIO led to fetal growth retardation. Moreover, maternal obesity inhibits placental nutrient transport efficiency. In particular down-regulation of the fatty acid transporters (CD36, FATP-1, and FATP-4) mRNA and protein expression may have an important role in the development of IUGR in the offspring of obese mothers. One of the most fundamental questions arising from the study is how DIO and DIR dams and their offspring differ in terms of their respective metabolic response to a HF diet. In further studies, the metabolic difference between the DIO and DIR dams should be investigated comprehensively. Growth and development differences in their offspring should be also explored. These differences may be fundamental to future understanding of the effect of diet on obesity and health.

## Figures and Tables

**Table 1:**
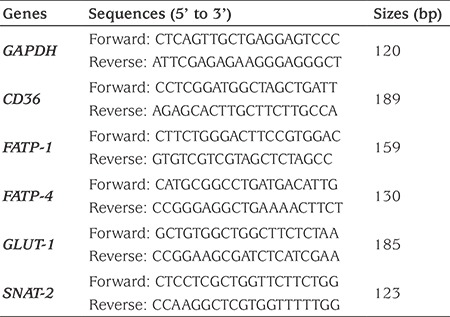
Oligonucleotide sequences and size of primers

## References

[1] Thrift AP, Callaway LK (2014). The effect of obesity on pregnancy outcomes among Australian Indigenous and non-Indigenous women. Med J Aust.

[2] Vinturache A, Moledina N, McDonald S, Slater D, Tough S (2014). Pre-pregnancy Body Mass Index (BMI) and delivery outcomes in a Canadian population. BMC Pregnancy Childbirth.

[3] Crane JM, Murphy P, Burrage L, Hutchens D (2013). Maternal and perinatal outcomes of extreme obesity in pregnancy. J Obstet Gynaecol Can.

[4] Bautista-Castaño I, Henriquez-Sanchez P, Alemán-Perez N, Garcia-Salvador JJ, Gonzalez-Quesada A, García-Hernández JA, Serra-Majem L (2013). Maternal obesity in early pregnancy and risk of adverse outcomes. PLoS One.

[5] Aune D, Saugstad OD, Henriksen T, Tonstad S (2014). Maternal body mass index and the risk of fetal death, stillbirth, and infant death: a systematic review and meta-analysis. JAMA.

[6] Yao R, Ananth CV, Park BY, Pereira L, Plante LA, Perinatal Research C (2014). Obesity and the risk of stillbirth: a population-based cohort study. J Obstet Gynecol.

[7] Persson M, Johansson S, Villamor E, Cnattingius S (2014). Maternal overweight and obesity and risks of severe birth-asphyxia-related complications in term infants: a population-based cohort study in Sweden. PLoS Med.

[8] Tenenbaum-Gavish K, Hod M (2013). Impact of maternal obesity on fetal health. Fetal Diagn Ther.

[9] Catalano PM, McIntyre HD, Cruickshank JK, McCance DR, Dyer AR, Metzger BE, Lowe LP, Trimble ER, Coustan DR, Hadden DR, Persson B, Hod M, Oats JJ, HAPO Study Cooperative Research Group (2012). The hyperglycemia and adverse pregnancy outcome study: associations of GDM and obesity with pregnancy outcomes. Diabetes Care.

[10] Hayes EK, Lechowicz A, Petrik JJ, Storozhuk Y, Paez-Parent S, Dai Q, Samjoo IA, Mansell M, Gruslin A, Holloway AC, Raha S (2012). Adverse fetal and neonatal outcomes associated with a life-long high fat diet: role of altered development of the placental vasculature. PLoS One.

[11] Fowden AL, Forhead AJ, Coan PM, Burton GJ (2008). The placenta and intrauterine programming. J Neuroendocrinol.

[12] Baumann MU, Deborde S, Illsley NP (2002). Placental glucose transfer and fetal growth. Endocrine.

[13] Day PE, Cleal JK, Lofthouse EM, Hanson MA, Lewis RM (2013). What factors determine placental glucose transfer kinetics. Placenta.

[14] Jansson T (2001). Amino acid transporters in the human placenta. Pediatr Res.

[15] Cleal JK, Lewis RM (2008). The mechanisms and regulation of placental amino acid transport to the human foetus. J Neuroendocrinol.

[16] Mackenzie B, Erickson JD (2004). Sodium-coupled neutral amino acid (System N/A) transporters of the SLC38 gene family. Pflugers Arch.

[17] Hatanaka T, Huang W, Martindale RG, Ganapathy V (2001). Differential influence of cAMP on the expression of the three subtypes (ATA1, ATA2, and ATA3) of the amino acid transport system A. FEBS Lett.

[18] Kazantzis M, Stahl A (2012). Fatty acid transport proteins, implications in physiology and disease. Biochim Biophys Acta.

[19] Cunningham P, McDermott L (2009). Long chain PUFA transport in human term placenta. J Nutr.

[20] Larque E, Demmelmair H, Klingler M, De Jonge S, Bondy B, Koletzko B (2006). Expression pattern of fatty acid transport protein-1 (FATP-1), FATP-4 and heart-fatty acid binding protein (H-FABP) genes in human term placenta. Early Hum Dev.

[21] Reynolds CM, Vickers MH, Harrison CJ, Segovia SA, Gray C (2015). Maternal high fat and/or salt consumption induces sex-specific inflammatory and nutrient transport in the rat placenta. Physiol Rep.

[22] Farley DM, Choi J, Dudley DJ, Li C, Jenkins SL, Myatt L, Nathanielsz PW (2010). Placental Amino Acid Transport and Placental Leptin Resistance in Pregnancies Complicated by Maternal Obesity. Placenta.

[23] Dube E, Gravel A, Martin C, Desparois G, Moussa I, Ethier-Chiasson M, Forest JC, Giguere Y, Masse A, Lafond J (2012). Modulation of fatty acid transport and metabolism by maternal obesity in the human full-term placenta. Biol Reprod.

[24] Zhu MJ, Ma Y, Long NM, Du M, Ford SP (2010). Maternal obesity markedly increases placental fatty acid transporter expression and fetal blood triglycerides at midgestation in the ewe. Am J Physiol Regul Integr Comp Physiol.

[25] Cifani C, Micioni Di Bonaventura MV, Pucci M, Giusepponi ME, Romano A, Di Francesco A, Maccarrone M, D’Addario C (2015). Regulation of hypothalamic neuropeptides gene expression in diet induced obesity resistant rats: possible targets for obesity prediction. Front Neurosci.

[26] Lin N, Cai DL, Jin D, Chen Y, Shi JJ (2014). Ginseng panaxoside Rb1 reduces body weight in diet-induced obese mice. Cell Biochem. Biophys.

[27] Rastelli VM, Akamine EH, Oliveira MA, Nigro D, Passaglia Rde C, Carvalho MH, Fortes ZB (2005). Influence of insulin on the microvascular response to inflammatory mediators in neonatal streptozotocin diabetic rats. Inflamm Res.

[28] Lee HC, Curry DL, Stern JS (1993). Tonic sympathetic nervous system inhibition of insulin secretion is diminished in obese Zucker rats. Obes Res.

[29] Zhou F, Chen H, Wang X, Yu P, Hu Y (2016). Hypoxia-induced regulation of placental REDD1 and mTOR was impaired in a rat model of estrogen-induced cholestasis. Arch Gynecol Obstet.

[30] Wang Y, Peng GQ, Jiang XQ, Dong LZ, Song WG, Zhang QL (2015). Comparison between Diabetic Rats’ Glucoses Measured by Two Types of Glucose Meters and the Full Automatic Biochemical Analyzer. aboratory Animal and Comparative Medicine.

[31] Subhasree N, Kamella A, Kaliappan I, Agrawal A, Dubey GP (2015). Antidiabetic and antihyperlipidemic activities of a novel polyherbal formulation in high fat diet/streptozotocin induced diabetic rat model. Indian J Pharmacol.

[32] Chen YH, Zhao M, Chen X, Zhang Y, Wang H, Huang YY, Wang Z, Zhang ZH, Zhang C, Xu DX (2012). Zinc supplementation during pregnancy protects against lipopolysaccharide-induced fetal growth restriction and demise through its anti-inflammatory effect. J Immunol.

[33] Perusse L, Bouchard C (2000). Gene-diet interactions in obesity. Am J Clin Nutr.

[34] Ricci MR, Levin BE (2003). Ontogeny of diet-induced obesity in selectively bred Sprague-Dawley rats. Am J Physiol Regul Integr Comp Physiol.

[35] Liu JM, Wang JX, Zheng L, Lian WG, Xu ZN, Liu SF (2041). Establishment of obesity prone and obesity resistant rat models and their metabolic study. Acta Nutrimenta Sinica.

[36] Crew RC, Waddell BJ, Mark PJ (2016). Maternal obesity induced by a ‘cafeteria’ diet in the rat does not increase inflammation in maternal, placental or fetal tissues in late gestation. Placenta.

[37] Aye IL, Rosario FJ, Powell TL, Jansson T (2015). Adiponectin supplementation in pregnant mice prevents the adverse effects of maternal obesity on placental function and fetal growth. Proc Natl Acad Sci U S A.

[38] Rosario FJ, Kanai Y, Powell TL, Jansson T (2015). Increased placental nutrient transport in a novel mouse model of maternal obesity with fetal overgrowth. Obesity (Silver Spring).

[39] Wallace JM, Horgan GW, Bhattacharya S (2012). Placental weight and efficiency in relation to maternal body mass index and the risk of pregnancy complications in women delivering singleton babies. Placenta.

[40] Lager S, Samulesson AM, Taylor PD, Poston L, Powell TL, Jansson T (2014). Diet-induced obesity in mice reduces placental efficiency and inhibits placental mTOR signaling. Physiol Rep.

[41] Larque E, Ruiz-Palacios M, Koletzko B (2013). Placental regulation of fetal nutrient supply. Curr Opin Clin Nutr Metab Care.

[42] Ortega-Senovilla H, Alvino G, Taricco E, Cetin I, Herrera E (2009). Enhanced circulating retinol and non-esterified fatty acids in pregnancies complicated with intrauterine growth restriction. Clin Sci (Lond).

